# Essential Oils as a Novel Anti-Biofilm Strategy Against *Salmonella* Enteritidis Isolated from Chicken Meat

**DOI:** 10.3390/microorganisms13102412

**Published:** 2025-10-21

**Authors:** Suzana Vidaković Knežević, Slobodan Knežević, Dubravka Milanov, Jelena Vranešević, Marko Pajić, Sunčica Kocić-Tanackov, Nedjeljko Karabasil

**Affiliations:** 1 Scientific Veterinary Institute “Novi Sad”, 21000 Novi Sad, Serbia; slobodan.knezevic@niv.ns.ac.rs (S.K.); dubravka@niv.ns.ac.rs (D.M.); jelenababic@niv.ns.ac.rs (J.V.); markopajic@niv.ns.ac.rs (M.P.); 2 Faculty of Technology Novi Sad, University of Novi Sad, 21000 Novi Sad, Serbia; suncicat@uns.ac.rs; 3 Faculty of Veterinary Medicine, University of Belgrade, 11000 Belgrade, Serbia; nedja@vet.bg.ac.rs

**Keywords:** *Salmonella* serovar, chicken meat, biofilm, oregano essential oil, cinnamon essential oil, rosemary essential oil, clove essential oil, thyme essential oils

## Abstract

*Salmonella* Enteritidis is a serious foodborne threat, being the most reported *Salmonella* serovar in the past several years. Biofilm formation contributes significantly to its persistence and resistance in food processing environments, making it harder to eliminate using conventional disinfectants. Recently, essential oils have emerged as promising natural alternatives due to their antimicrobial and anti-biofilm properties. In this study, the biofilm-forming ability of *Salmonella* Enteritidis, isolated from chicken meat, was evaluated under various nutrient conditions and temperatures. Furthermore, the anti-biofilm activity of essential oils derived from oregano, cinnamon, rosemary, clove, and thyme was assessed against strong and moderate biofilms formed by *Salmonella* Enteritidis. The isolates demonstrated the capacity to form biofilms in tryptic soy broth, meat broth, and Luria–Bertani broth at 37 °C, 15 °C, and 5 °C. All selected essential oils, at their minimum bactericidal concentrations, effectively reduced preformed biofilms by between 36.98% to 74.83%. The destructive effect of essential oils on *Salmonella* Enteritidis bacterial cells was further confirmed through scanning electron microscopy analysis. In conclusion, the selected essential oils exhibited promising anti-biofilm potential and may serve as effective natural agents for controlling biofilm-associated contamination by *Salmonella* Enteritidis.

## 1. Introduction

The latest European Union One Health report [1] presents salmonellosis as the second most common foodborne infection in humans. An increase of 16.90% in confirmed cases was reported in 2023, compared with 2022. *Salmonella* Enteritidis was the most reported *Salmonella* serovar, with 70.80% of human infections. The most *Salmonella*-contaminated food categories were of poultry origin (30.80%), including fresh meat, mechanically separated meat, minced meat, meat preparations, and meat products.

The *Salmonella* species, although an intestinal pathogen, is well adapted to food processing environments in the form of biofilm, from where it poses a potential health risk for society [2]. The formation of *Salmonella* biofilm consists of the typical biofilm-forming stages: initial reversible attachment of planktonic cells, irreversible attachment, biofilm growth and maturation, detachment and dispersal of planktonic cells [3]. *Salmonella* is able to form biofilms on several different materials used in the food industry, including plastic, stainless steel, rubber, and glass [2,3]. The biofilm forms a protective shield for *Salmonella* cells, allowing them to be persistent and to contaminate new surfaces. The eradication of *Salmonella* biofilms, through standard cleaning procedures using chemical disinfectants, is challenging [4]. Adding to concerns about the toxic effects of synthetic chemicals, the search for new green alternatives has become important. Although known for being ancient, essential oils (EOs), which are secondary metabolites of different parts of plants (roots, bark, leaves, flowers, seeds), have become a new control strategy against biofilms formed on food-contact surfaces [5]. Different mechanisms are involved in EOs’ action against biofilms, including the inhibition of matrix formation, the influence on the quorum sensing (QS) system, and the effects on gene expression [6]. The mechanisms of EOs against biofilms are influenced by their complex chemical composition. Essential oils can contain between nine and sixty identified chemical compounds [7,8], with the major compounds comprising up to 88% of the total composition [8]. The EO compounds act synergistically, simultaneously targeting several sites within the bacterial cell. This ability to affect various metabolic pathways and cellular structures, with different compounds, makes it more difficult for bacteria to develop resistance against EOs [9,10,11]. Essential oils rich in phenolic compounds (carvacrol, eugenol, and thymol), aldehydes (cinnamaldehyde), and terpenes (α-pinene and p-cymene), such as oregano, cinnamon, rosemary, clove, and thyme, exhibit strong antibacterial [8,10,12,13] and anti-biofilm activities [5,8,12,14]. Carvacrol, eugenol, and thymol primarily act on the bacterial cytoplasmic membrane, disrupting its integrity and interfering with the active transport and exchange of materials within the cell. Additionally, eugenol can inactivate vital enzymes. Alpha-pinene and p-cymene have been shown to inhibit protein function and DNA synthesis, as well as to cause disruption or rupture of the bacterial cell membrane [15,16]. Cinnamaldehyde interferes with the QS system and has an impact on cell communication in the biofilm [17].

However, the effectiveness of EOs against biofilms depends on the surface material on which the biofilm is formed, the concentration of EO, exposure time, the type of biofilm (single-species or multi-species), and others [5]. In this study, we aimed to determine the ability of four *S.* Enteritidis isolated from chicken meat to form biofilms in different conditions of nutrient media (tryptone soy broth (TSB), meat broth (MB), and Luria–Bertani broth (LBB)) and temperature (37 °C, 15 °C, and 5 °C). Additionally, this study aimed to evaluate the effect of selected commercially available EOs on preformed *S.* Enteritidis biofilms, as well as to observe morphological changes in bacterial cells after EO treatment using scanning electron microscopy (SEM).

## 2. Materials and Methods

### 2.1. Bacterial Isolates

*S.* Enteritidis isolates, SE53, SE56, SE132, and SE144, were isolated from chicken meat originating from a slaughterhouse in the Kraljevo region of Serbia and identified using standard ISO methods 6579-1 [18], and ISO 6579-3 [19], respectively. In brief, 25 g of chicken meat sample was homogenized with a Stomacher (Mayo International SRL, Novate Milanese, Italy) for 2 min in 225 mL buffered peptone water (Biokar Diagnostics, Beauvais, France) and incubated at 37 °C for 18 h. Then, aliquots of 0.1 mL and 1 mL were transferred to the Rappaport–Vassiliadis soya peptone (RVS) (Biokar Diagnostics, Beauvais, France) and Muller–Kauffmann tetrathionate–novobiocin broth (MKTTn) (Biokar Diagnostics, Beauvais, France), respectively. The RVS was incubated at 41.5 °C, while the MKTTn was incubated at 37 °C for 24 h. A loop-full volume (10 µL) from both RVS and MKTTn were inoculated on xylose lysine deoxycholate (CM0469, Oxoid, Basingstoke, UK) and *Salmonella* differential agar (HiMedia Laboratories Pvt. Ltd., Mumbai, India), inverted, and incubated at 37 °C for 24 h.

Serotyping was carried out using O:9, H:g, H:m, H:q, H:s, and O:46 antisera (SSI Diagnostica A/S, Hillerød, Denmark), following the schematic overview for serotyping given in ISO 6579-3. All *S.* Enteritidis isolates were stored at −80 °C until analysis.

Bacterial suspensions were prepared by incubating the *S.* Enteritidis isolates (SE53, SE56, SE132, and SE144) in 3000 μL of tryptic soy broth (TSB) (Oxoid, Basingstoke, UK) overnight at 37 °C. Then, aliquots of 100 μL were diluted in 3900 μL TSB.

### 2.2. Essential Oils

Five commercially available EOs (TerraCo d.o.o., Novi Sad, Serbia) were selected for this study. The EOs of oregano, cinnamon, rosemary, clove, and thyme are recognized as GRAS (Generally Recognized as Safe) by the Food and Drug Administration [20], which supports their potential applicability in food-related settings. All EOs were stored in dark glass bottles at 4 °C until use.

A gas chromatograph GC 7890B, coupled with an MS 5977A mass spectrometer (Agilent Technologies, Santa Clara, CA, USA), was used to identify the compounds in each EO [21]. The relative amounts of the main compounds were determined using the area normalization method, without applying response factors. Main compound percentages were reported based on GC–MS analysis and reflect the proportion of main compounds relative to the total of all compounds detected in the chromatogram. The minimal bactericidal concentrations (MBCs) were determined using the broth microdilution method, where the lowest concentration without visible growth of *S.* Enteritidis was considered as the MBC [22]. The details of the EOs are given in Table 1.

### 2.3. Biofilm Formation

Biofilm formation was performed by applying the indirect quantitative method on polystyrene microtiter plates using the crystal violet test [8,23]. Briefly, aliquots of 200 µL of bacterial suspension prepared in different broths, including the tryptic soy broth (TSB) (Oxoid, Basingstoke, UK), meat broth (MB) (Oxoid, Basingstoke, UK), and Luria–Bertani broth (LBB) (Oxoid, Basingstoke, UK) were incubated for 48 h at three temperatures (37 °C, 15 °C, and 5 °C). Then, the wells were washed using physiological saline and dried at room temperature. The adhered *Salmonella* cells were fixed (250 μL/well of 96% ethanol (Reahem, Srbobran, Serbia)) and dyed (0.3% crystal violet (Fluka, Sigma-Aldrich, Darmstadt, Germany)). Then, the wells were rinsed using tapped water and air dried. The crystal violet dye bound to the biofilm cells was solubilized by adding ethanol (250 μL/well of 96% ethanol) prior to recording its optical density (OD_550_) (ASYS Expert Plus Microtitration Reader, Biochrom, Cambridge, UK). The *Salmonella* isolates were classified as strong, moderate, weak, and non-biofilm producer [23] based on the average value of the measured optical densities (ODs). The OD cut-off values were defined as the mean OD of the negative controls of each broth plus three standard deviations.

### 2.4. Biofilm Reduction

All strong and moderate *S.* Enteritidis biofilm producers, formed according to Section 2.3., were further treated with the MBCs of oregano, cinnamon, rosemary, clove, and thyme EOs. In brief, after washing microtiter plate wells with physiological saline, the adhered *S.* Enteritidis cells were treated with 200 µL of EO solution in TSB and incubated for 48 h at the temperature of biofilm formation (37 °C and 15 °C). After 48 h, the wells were washed using physiological saline (3 × 250 μL/well) and dyed using 0.3% of crystal violet solution (Fluka, Sigma-Aldrich, Darmstadt, Germany). Following 20 min of staining, the dye bound to the bacterial cells was rinsed with the tapped water, and the wells were once again air dried. Next, 250 μL/well of 96% ethanol was added to each well to solubilize the crystal violet for optical density measurement. The optical density was measured using ASYS Expert Plus Microtitration Reader (Biochrom, Cambridge, UK). The inhibition percentages were calculated for each EO by Formula (1) [24]:

[(OD_GROWTHCONTROL_ − OD_SAMPLE_)/OD_GROWTH CONTROL_] × 100, (1)

### 2.5. Scanning Electron Microscopy

Scanning electron microscopy (SEM) was performed on stainless steel to observe the effect of oregano, cinnamon, rosemary, clove, and thyme EOs on the *S.* Enteritidis isolate SE144 which formed a strong biofilm. The stainless steel (SS 304) coupons were covered with 100 µL of SE144 solution and incubated for 3 h at 37 °C. After incubation, the coupons were washed with physiological saline. Prior to 24 h of incubation at 37 °C aliquots (2000 µL) of LBB were used to cover the coupons. After incubation, the non-adhered SE144 cells were washed with physiological saline. The ½ MBC EO solutions were added onto coupons and incubated for another 24 h at 37 °C. Then, the coupons were washed with physiological saline and fixed overnight at 5 °C with 4% glutaraldehyde (Centrohem, Stara Pazova, Serbia). Once again, the coupons were washed with physiological saline, followed by 5 min of graded ethanol dehydration (30%, 50%, 60%, 70%, and 90%). Additionally, coupons were left (3 × 10 min) in 96% ethanol prior to drying, gold coating (BAL-TEC SCD 005, Balzers, Liechtenstein) and observation with a scanning electron microscope (JMS SEM 6460 LV, Tokyo, Japan).

### 2.6. Statistical Analysis

Statistical analysis was conducted by analysis of variance (ANOVA), while the means were compared by Duncan’s test using statistical software R version 3.2.2 (R Foundation for Statistical Computing, Vienna, Austria). The differences were considered as significant if *p* < 0.05.

## 3. Results

### 3.1. Salmonella Enteritidis Biofilm Formation

To classify the biofilms, the following cut-off values were established: TSB (37 °C) = 0.108; MB (37 °C) = 0.133; LBB (37 °C) = 0.123; TSB (15 °C) = 0.173; MB (15 °C) = 0.167; LBB (15 °C) = 0.159; TSB (5 °C) = 0.150; MB (5 °C) = 0.161; LBB (5 °C) = 0.113.

The effect of different nutrients (TSB, MB, LBB) and temperatures (37 °C, 15 °C, and 5 °C) on the biofilm formation of the four *S.* Enteritidis isolates derived from chicken meat are presented in Table 2. Under different conditions of media and temperature, one *S.* Enteritidis isolate was classified as a strong biofilm producer (SE144), six as moderate, twenty-six as weak, and three as non-biofilm producers.

### 3.2. Salmonella Enteritidis Biofilm Reduction

Table 3 presents the results of the effects of various EOs on preformed strong and moderate *S.* Enteritidis biofilms. The reduction in preformed *S.* Enteritidis biofilms varied from 36.98% to 74.83% when treated with cinnamon and oregano EOs, respectively. Cinnamon EO exhibited the broadest spectrum of activity in reducing the biomass of preformed *S.* Enteritidis biofilms under different incubation conditions, showing the greatest variability in effect (35.47%), whereas rosemary EO demonstrated the smallest differences (25.68%). No statistically significant (*p* > 0.05) effect of EOs on preformed *Salmonella* biofilms was observed during the incubation of isolate SE56 in TSB at 37 °C and isolate SE132 in LBB at 37 °C.

### 3.3. Scanning Electron Microscopy Observation 

**Figure 1:**
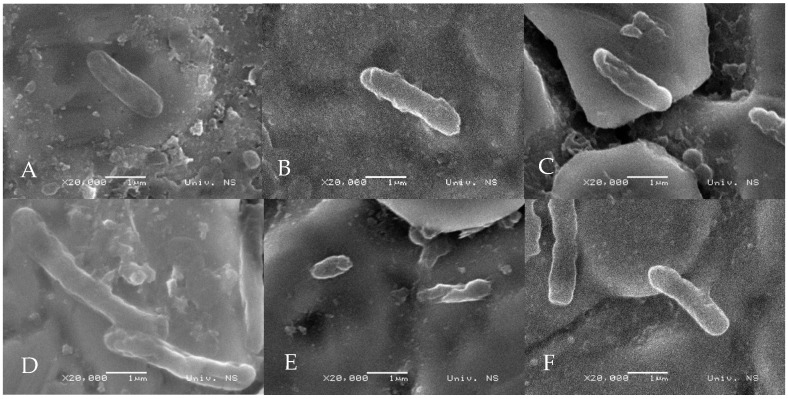
The SEM micrographs show the *S.* Enteritidis cells: (**A**) untreated and treated with (**B**) oregano (0.23 µL/mL), (**C**) cinnamon (0.23 µL/mL), (**D**) rosemary (1.78 µL/mL), (**E**) clove (0.45 µL/mL), and (**F**) thyme (0.45 µL/mL) essential oils.

Figure 1 shows SEM micrographs at ×20,000 magnification, revealing the structure of the untreated *S.* Enteritidis cell (Figure 1A), and the *S.* Enteritidis cells treated with oregano (Figure 1B), cinnamon (Figure 1C), rosemary (Figure 1D), clove (Figure 1E), and thyme (Figure 1F) EOs. The untreated *S.* Enteritidis cell has its typical rod-shaped structure, smooth surface, and well-defined boundaries. In contrast, observable structural alterations in the *S.* Enteritidis cell, caused by the treatment, were imaged. This includes abnormalities in cell morphology (such as shrinkage, deformation, or irregular shapes) and disruption of cell wall or membrane.

## 4. Discussion

In this study, we demonstrated the ability of four *S.* Enteritidis isolates, previously found in chicken meat, to form biofilms in different nutrient (TSB, MB, and LBB) and temperature (37 °C, 15 °C, and 5 °C) conditions. Additionally, the anti-biofilm effect of oregano, cinnamon, rosemary, clove, and thyme EOs was evaluated.

It is generally accepted that nutrient-poor media have a more favorable effect on the formation of *Salmonella* spp. biofilms. According to Stepanović et al. [25], 20-fold dilution TSB proved to be the most effective medium for biofilm development in the majority (72.9%) of the tested *Salmonella* spp. isolates. The remaining 27.1% formed more substantial biofilms in richer media, such as the Brain Heart Infusion broth (BHI), TSB, and MB. This observation implies that biofilm formation could serve as a survival strategy in nutrient-limited environments [26]. The TSB and LBB are nutrient-rich media, and this study shows that eight weak and three moderate *S.* Enteritidis biofilms were formed in both media. However, the LBB media supported the formation of one strong biofilm. On the contrary, the TSB and LBB were not conductive for **Salmonella* enterica* biofilm formation compared to the 20-fold TSB and LBB without NaCl [27]. Previous research has shown that TSB is suitable for the formation of biofilms by various *Salmonella* strains, including *S.* Enteritidis, *S.* Typhimurium, *S.* Infantis, *S.* Virchow, *S.* Derby, *S.* Agona, and *S.* Newport [28]. Although the culture media used in this study, including MB, TSB, and LBB, do not fully replicate the complex environment found in poultry processing facilities, they were selected because they support robust biofilm formation in vitro and are commonly used in biofilm research. These nutrient-rich media enable the evaluation of biofilm development under controlled laboratory conditions. Additionally, these media serve as standardized models to study bacterial behavior relevant to the food industry, even though they cannot fully mimic all in vivo conditions.

The temperature of 37 °C was chosen as the optimal growth temperature for *S.* Enteritidis, while lower temperatures were used to simulate conditions in poultry slaughterhouse facilities (15 °C) and refrigeration storage (5 °C). While regulations recommend not exceeding 12 °C ambient temperature, 15 °C is sometimes observed in practice in the evisceration area of poultry slaughterhouses [29]. The strongest biofilm formation was observed at 37 °C, followed by 15 °C. However, all tested *S.* Enteritidis isolates demonstrated the ability to form biofilms at 5 °C, which is a temperature that reflects typical household refrigeration conditions [30], potentially increasing the risk of cross-contamination during food storage. The *S.* Enteritidis isolates from our study most likely formed stronger biofilms at 37 °C and 15 °C due to more favorable conditions for bacterial metabolic activity and growth. The formation of biofilms is an active, energy-dependent process that requires cell division, production of EPS, and QS, all of which are reduced at low temperatures [31]. At 37 °C, bacteria grow optimally, promoting rapid attachment and biofilm maturation. Although 15 °C is suboptimal for growth, it still allows sufficient metabolic activity to support biofilm development, particularly under stress. In contrast, at 5 °C, metabolic rates are drastically reduced, leading to minimal biofilm formation.

De Oliveira et al. [32] examined the biofilm formation ability of 174 *Salmonella* spp. isolates on PVC (polyvinyl chloride) surfaces at different temperatures, with 35 °C (65.5%) being the most favorable compared to 28 °C (44.8%), 20 °C (39.7%), and 16 °C (44.8%). All isolates that formed biofilm were classified as poor biofilm producers. The authors believe that, although *Salmonella* was not identified, most of the isolates belong to *S.* Enteritidis, given that they were isolated from raw chicken meat. On the other hand, in the study by Stepanović et al. [33], 30 °C was statistically more favorable for biofilm formation in 29 *S.* Enteritidis isolates than 37 °C and ~22 °C during a 24 h incubation. However, by extending the incubation to 48 h, the highest amount of biofilm formation was achieved at a temperature of ~22 °C. A similar observation was made with 40 **Salmonella* enterica* isolates originating from pig slaughterhouses, which statistically formed biofilms better at a temperature of 22 °C compared to 35 °C [34].

The EOs were able to reduce the preformed *S.* Enteritidis biofilms in the range from 36.98% to 74.83%. The results indicate the potential of EOs to penetrate biofilms and kill the protected bacterial cells. The removal of established biofilms involves targeting bacterial cells that are embedded within a complex EPS matrix, which serves as a protective barrier against antimicrobial agents [5]. Therefore, evaluating the activity of EOs against mature biofilms is crucial, as it provides insight into the ability of the EOs to penetrate the EPS matrix and act on surface-attached bacterial cells. This approach better reflects food-related environmental conditions, where biofilms are typically well-developed and more resistant to treatment. However, it is important to distinguish between mature biofilms formed on food-contact surfaces (e.g., equipment, packaging) and those potentially forming directly on raw meat. In the latter case, the presence of mature biofilms may directly compromise the sensory and microbiological quality of the product, raising concerns not only about safety but also about product acceptability. While the current study focuses on the efficacy of EOs against mature biofilms on surfaces, it does not address the impact of such treatment on the quality of raw meat. Moreover, the prevention of initial bacterial attachment to meat surfaces may represent a more appropriate strategy for application in food matrices. This distinction highlights a limitation of the present study and underscores the need for future research to evaluate the dual impact of EO-based treatments, both in terms of microbial reduction and preservation of food quality.

The anti-biofilm activity of EOs is largely influenced by the presence of their main chemical compounds. Carvacrol, the main compound of oregano EO, primary acts on the outer membrane of bacterial cells. However, it is believed that its actual site of action is the cytoplasmic membrane, disrupting normal membrane function and increasing membrane permeability [9,35,36]. Similarly, thymol, the main compound of thyme EO, acts on bacterial cells through the same mechanism, as a proton exchanger leading to a reduction in the transmembrane gradient, collapse of the proton pump, inhibition of the respiratory chain, oxidation, and loss of cellular components [37,38,39,40,41]. EOs exhibit strong anti-biofilm activity through multiple mechanisms. They interfere with bacterial adhesion proteins, inhibit motility by affecting flagellar assembly and QS-regulated pathways, and disrupt biofilm maturation by degrading the EPS matrix and altering its composition. Certain EOs, such as cinnamon EO, weaken biofilm structure by modifying protein profiles [42]. The synergistic effect of EO compounds on biofilm is manifested through the dual action of their hydrophilic and hydrophobic moieties. The hydrophilic groups (carvacrol and thymol) facilitate penetration through the biofilm’s EPS matrix, whereas the hydrophobic groups enable interaction with, and disruption of, bacterial membrane lipids [5]. This causes destabilization of the bacterial cell envelope, leading to increased membrane permeability, cytoplasmic leakage, metabolic dysfunction, and ultimately, cell death. In addition to physical disruption, EOs also influence gene expression, enzyme activity, and proteomic profiles associated with biofilm development and virulence. Furthermore, many EOs interfere with QS pathways. These combined actions highlight the broad-spectrum potential of EOs as effective anti-biofilm agents [42].

As an adaptive mechanism to maintain optimal membrane function and structure, bacterial cells exposed to carvacrol are thought to alter the fatty acid composition of their membranes [43]. The anti-biofilm activity of cinnamon EO is attributed to the presence of cinnamaldehyde, which is an aromatic aldehyde that inhibits the synthesis of essential bacterial enzymes and/or causes damage to the bacterial cell wall [44,45]. Both α-pinene and borneol, which are the two main compounds of rosemary EO, have previously shown inhibition of microorganisms in low concentrations [46]. Eugenol, the main compound of clove EO, has the ability to irreversibly damage the cell membrane and cellular structures, leading to the leakage of biomacromolecules (ATP, DNA, ions, and proteins) and intracellular enzymes. Consequently, the physiological activity of the cells decreases, resulting in cell death [15,47]. The strong antibacterial activity of thyme EO can be attributed to its high content of *p*-cymene (40.91%) and thymol (40.36%). The hydrophobic compound *p*-cymene, a precursor of carvacrol, causes swelling of the cytoplasmic membrane [48], while thymol increases its permeability [35] and leads to the loss of intracellular components [49].

In the SEM assay, ½ MBC of the EOs was used to treat the biofilms. This sub-lethal concentration was chosen to evaluate the effects of the EOs on biofilm morphology and bacterial cell integrity without causing complete bacterial death. The changes in *S.* Enteritidis cell morphology observed via SEM micrographs in this study, resulting from the effects of selected EOs, are consistent with findings from other authors [8,12,14,50,51], who also reported loss of cell shape and structural integrity, followed by cell death. This may be a consequence of extensive leakage of intracellular contents or the induction of cell self-lysis [38].

Considering the results of this study and the fact that applied EOs, such as oregano, cinnamon, rosemary, clove, and thyme, are recognized as GRAS [20], their application in controlling biofilms in poultry processing environments or on raw meat surfaces could contribute to food safety. However, a major limitation of their practical use lies in the strong aroma and flavor of EOs, which may negatively affect the organoleptic properties of meat products. Strategies such as the encapsulation of EOs or their incorporation into active packaging systems have been proposed to overcome this issue [52]. These approaches could enable a controlled release of EOs, thereby minimizing their sensory impact while helping to extend shelf life and maintain the microbiological quality and safety of poultry meat.

On the other hand, one of the main limitations of this study is the use of only a single concentration of EOs, which does not allow for an assessment of the dose-dependent effects on *S.* Enteritidis biofilms formed under different conditions of media and temperature. Additionally, the study evaluated EOs as whole, complex mixtures, without investigating their individual chemical compounds. This limits the ability to identify specific bioactive compounds responsible for the observed anti-biofilm activity and hinders a deeper understanding of their mechanisms of action.

Future research should aim to include the fractionation and testing of individual components to better characterize the active substances and explore potential synergistic interactions. Moreover, validation through in situ trials within food industry settings is essential to assess their practical applicability as natural disinfectant strategies.

## 5. Conclusions

*S.* Enteritidis remains a significant foodborne pathogen, with strong biofilm-forming potential under various environmental conditions, including different nutrient media and temperatures. The findings of this study demonstrate that oregano, cinnamon, rosemary, clove, and thyme EOs exhibited notable reduction in preformed biofilms, with effectiveness reaching up to 74.83% at their minimum bactericidal concentrations. SEM further confirmed the disruptive impact of these EOs on the cell morphology of *S.* Enteritidis, indicating the membrane damage and structural collapse of biofilm-embedded cells. These results suggest that selected EOs hold considerable promise as natural, effective agents for the control of *S.* Enteritidis biofilms in food processing environments. Given their efficacy, especially at 37 °C and 15 °C, these EOs could be further explored for use in surface disinfectants, cleaning formulations, or coating materials in poultry processing facilities.

## Figures and Tables

**Table 1:** Main chemical compounds (%) and minimal bactericidal concentrations (µL/mL) of selected essential oils.

Essential Oils	Latin Name	Main Compounds (%) *	Minimal Bactericidal Concentrations (MBC) (µL/mL) *			
			SE53	SE56	SE132	SE144
Oregano	*Origanum vulgare*	carvacrol (81.00%)	0.09	0.18	0.45	0.45
Cinnamon	*Cinnamomum zeylanicum* Nees	cinnamaldehyde (74.93%)	0.89	0.45	0.45	0.45
Rosemary	*Rosmarinus officinalis*	α-pinene (28.23%), borneol (24.87%)	1.78	1.78	0.45	3.56
Clove	*Syzygium aromaticum* L.	eugenol (85.14%)	0.89	0.45	0.89	0.89
Thyme	*Thymus vulgaris*	p-cymene (40.91%), thymol (40.36%)	0.45	1.78	0.89	0.89

* Results from Vidaković Knežević et al. [21].

**Table 2:** *Salmonella* Enteritidis biofilm formation under different conditions of temperatures and nutrient media on the polystyrene surface.

Temperature (°C)	Nutrient Media	SE53	SE56	SE132	SE144
37	TSB	0.282 ± 0.061 **	0.291 ± 0.073 **	0.136 ± 0.013 *	0.192 ± 0.034 *
	MB	0.185 ± 0.005 *	0.215 ± 0.018 *	0.215 ± 0.035 *	0.174 ± 0.010 *
	LBB	0.225 ± 0.014 *	0.464 ± 0.062 **	0.481 ± 0.092 **	0.530 ± 0.084 ***
15	TSB	0.188 ± 0.010 *	0.186 ± 0.011 *	0.445 ± 0.087 **	0.163 ± 0.007 °
	MB	0.173 ± 0.008 *	0.210 ± 0.015 *	0.191 ± 0.014 *	0.162 ± 0.007 °
	LBB	0.240 ± 0.051 *	0.196 ± 0.016 *	0.406 ± 0.051 **	0.161 ± 0.017 *
5	TSB	0.247 ± 0.018 *	0.222 ± 0.009 *	0.187 ± 0.010 *	0.159 ± 0.011 *
	MB	0.199 ± 0.025 *	0.208 ± 0.020 *	0.214 ± 0.025 *	0.145 ± 0.006 °
	LBB	0.185 ± 0.015 *	0.157 ± 0.014 *	0.170 ± 0.014 *	0.128 ± 0.009 *

Values are expressed as mean OD_550_ ± SD. Classification of biofilm producers: strong (***), moderate (**); weak (*); non (°).

**Table 3:** *Salmonella* Enteritidis biofilm reduction (%) after exposure to the MBC of selected essential oils.

Isolates	Conditions	Essential Oils				
		Oregano	Cinnamon	Rosemary	Clove	Thyme
SE53	TSB/37 °C	52.30 ^b^	45.09 ^a^	46.10 ^ab^	48.79 ^ab^	43.91 ^a^
SE56	TSB/37 °C	53.81 ^a^	48.85 ^a^	53.09 ^a^	51.49 ^a^	48.42 ^a^
SE56	LBB/37 °C	48.33 ^ab^	44.97 ^a^	50.14 ^ab^	55.62 ^b^	50.57 ^ab^
SE132	LBB/37 °C	48.48 ^a^	42.33 ^a^	40.07 ^a^	44.68 ^a^	44.77 ^a^
SE132	TSB/15 °C	74.83 ^b^	72.45 ^b^	65.75 ^a^	71.57 ^b^	72.79 ^b^
SE132	LBB/15 °C	71.61 ^c^	60.14 ^b^	54.99 ^a^	70.83 ^c^	69.05 ^c^
SE144	LBB/37 °C	47.06 ^b^	36.98 ^a^	50.86 ^bc^	58.18 ^c^	52.47 ^bc^

Values with different small letters (a, b, c) in superscript, within the same row, indicate statistically significant differences (*p* < 0.05).

## Data Availability

The original contributions presented in the study are included in the article, and further inquiries can be directed at the corresponding author.
